# miR-92a-3p Exerts Various Effects in Glioma and Glioma Stem-Like Cells Specifically Targeting CDH1/β-Catenin and Notch-1/Akt Signaling Pathways

**DOI:** 10.3390/ijms17111799

**Published:** 2016-10-27

**Authors:** Hang Song, Yao Zhang, Na Liu, Sheng Zhao, Yan Kong, Liudi Yuan

**Affiliations:** 1 State Education Ministry’s Key Laboratory of Developmental Genes and Human Diseases, Southeast University, No. 2 Sipailou Road, Nanjing 210096, China; tosonghang@sina.com (H.S.); zhangyao_seu2014@163.com (Y.Z.); liunabeauty@163.com (N.L.); 2 Department of Biochemistry and Molecular Biology, Medical School of Southeast University, No. 87 Dingjiaqiao Road, Nanjing 210009, China; windupzs@gmail.com (S.Z.); kongyancn@gmail.com (Y.K.)

**Keywords:** glioma, glioma stem-like cells, miR-92a-3p, CDH1, Notch-1

## Abstract

MicroRNAs (miRNAs) are implicated in the regulation of tumor progression and stemness of cancer stem-like cells. Recently, miR-92a-3p was reported to be up-regulated in human glioma samples. Nevertheless, the precise role of miR-92a-3p in glioma cells and glioma stem-like cells (GSCs) has not been fully elucidated. It is necessary to clarify the function of miR-92a-3p in glioma and GSCs to develop novel therapeutic approaches for glioma patients. In the present study, we applied methyl-thiazolyl-tetrazolium (MTT) assay and Transwell assay to measure the proliferation rate and metastatic potential of glioma cells. Meanwhile, the self-renewal ability of GSCs was detected by tumor sphere formation assay. The results revealed that down-regulation of miR-92a-3p suppressed the glioma cell malignancy in vitro. Moreover, knockdown of miR-92a-3p led to a reduction of tumorgenesis in vivo. Interestingly, we also observed that up-regulation of miR-92a-3p could inhibit the stemness of GSCs. Subsequent mechanistic investigation indicated that cadherin 1 (CDH1)/β-catenin signaling and Notch-1/Akt signaling were the downstream pathways of miR-92a-3p in glioma cells and GSCs, respectively. These results suggest that miR-92a-3p plays different roles in glioma cells and GSCs through regulating different signaling pathways.

## 1. Introduction

Glioblastoma (GBM) is the most prevalent and aggressive primary type of brain tumor characterized with highly morbidity and mortality [1]. Although major progresses and advancements have been made in the surgical resection, postoperative chemotherapy, and radiation, the prognosis for malignant gliomas remains dismal with an average survival of roughly one year after diagnosis [2,3,4]. It is also revealed that a small subpopulation of cancer cells, termed glioma stem-like cells (GSCs), exist in glioma cells [5]. These GSCs are regarded as the main reason for tumor recurrence, tumor infiltration and sensitivity to conventional therapies [6,7]. However, the molecular mechanism involved in the progression of glioma and GSCs remains unclear, elucidation is essential for the designation of novel therapeutic approaches to GBM.

MicroRNAs (miRNAs) are a group of small, and highly conserved non-coding RNA molecules of nearly 20–25 nucleotides which regulate gene expression by promoting degradation of mRNA and/or inhibition of protein translation mainly by direct binding to the 3′ untranslated region (UTR) of downstream mRNAs at the posttranscriptional level [8,9]. Numerous researches have provided evidence that miRNAs are implicated in the tumorgenesis, carcinogenesis, and tumor angiogenesis by functioning as tumor promoters or as tumor suppressors [10,11]. In addition, miRNAs were also found to be related to the differentiation and self-renewal and of stem cells [12]. For example, let-7, one of the first identified miRNA, not only inhibits the malignancy of breast cancer, but also suppresses the self-renewal ability of breast cancer stem-like cells [13]. By direct regulating the key genes of glioma development, miR-218 suppresses the invasion and self-renewal of glioma [14].

miR-92a-3p belongs to the miR-17-92 family that plays a critical role in modulating cell viability, apoptosis and metastasis [15,16]. It has been reported that miR-92a-3p act like an onco-miR in human colorectal cancer cells through regulating the PTEN/PI3K/AKT signaling pathway [17]. More importantly, miR-92a-3p is also reported to be up-regulated in human glioma samples and regulates glioma apoptosis through targeting Bim [18].

In this study, we concentrated our intention on miR-92a-3p and identified that down-regulation of miR-92a-3p inhibited the malignancy of glioma cells significantly in vitro as well as impeded the formation of xenograft tumors in vivo. Interestingly, up-regulation of miR-92a-3p decreased the stem-like traits of glioma derived GSCs. Mechanically, we demonstrated that miR-92a-3p targeted CDH1/β-catenin signaling in glioma cells, while Notch-1/Akt signaling was the downstream pathway of miR-92a-3p in GSCs. These findings suggest that miR-92a-3p functions through targeting various pathways in glioma cells and GSCs.

## 2. Results

### 2.1. Depleting Endogenous miR-92a-3p Inhibits the Proliferationand Metastasis of Glioma Cells In Vitro While Preventing Xenograft Growth of Glioma In Vivo

**Figure 1:**
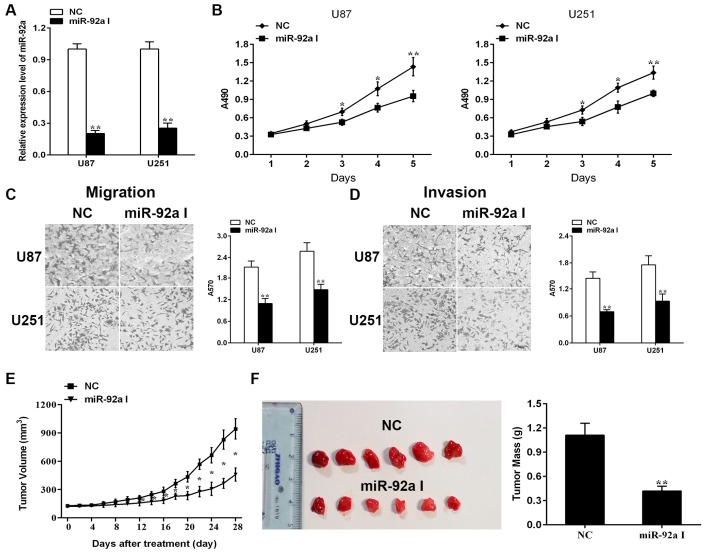
Effect of miR-92a-3p on the malignancy of glioma cells in vitro and in vivo. (**A**) Quantitative reverse transcription-polymerase chain reaction (qRT-PCR) analysis of miR-92a-3p expression in glioma cells 48h-post transfection with miR-92a-3p I; (**B**) The rates of cell viability were quantified using a methyl-thiazolyl-tetrazolium (MTT) assay; Cell migration (**C**) and invasion (**D**) was detected by Transwell assay; (**E**) Tumor volumes were measured every two days during treatment; (**F**) Mice were sacrificed 28 days following miR-92a-3p I injection and the tumors were weighted. All Transwell images were photographed at 100× original magnification. Each bar represents the mean of three independent experiments. * indicates *p* < 0.05; ** indicates *p* < 0.01. Scale bar represents 200 µm.

Previous results inhibited that miR-92a-3p was aberrant up-regulated not only in human glioma cell lines but also in glioma samples [18], suggesting that down-regulation of miR-92a-3p may exert antitumor effects in glioma. To test this hypothesis, miR-92a-3p I was uesd to deplete endogenous miR-92a-3p in U87 and U251 cell lines. The relative expression of miR-92a-3p was detected with Quantitative reverse transcription-polymerase chain reaction (qRT-PCR). miR-92a-3p expression level was decreased by up to 80% in two glioma cell lines post-transfected with miR-92a-3p I (Figure 1A). Then the role of miR-92a-3p in glioma cell proliferation was identified by methyl-thiazolyl-tetrazolium (MTT) assay. As illustrated in Figure 1B, the proliferation rate was significantly lower from day 3 to 5 in the miR-92a-3p I group than that in the negative control (NC) group. Transwell assay implied that inhibition of miR-92a could markedly attenuate both glioma cell migration and invasion. When compared with the group of negative control, the number of migrated cells was decreased to roughly 51.3% in U87 cells and to nearly 57.4% in U251 cells in the miR-92a-3p I group (Figure 1C). Similarly, the number of invaded cells was also markedly reduced in glioma cells after treatment with miR-92a-3p I (Figure 1D). The above results suggested that miR-92a-3p may play a part in the maintenance of glioma proliferation and metastasis in vitro.

To determine the function of miR-92a-3p in glioma carcinogenesis, we assessed the role of miR-92a-3p inhibition on tumorgenesis in vivo. When the mean tumor volume reached about 100 mm^3^, 200 pmol NC or miR-92a-3p I in 10 µL Lipofectamine 2000 were treated with all nude mice every two days for 28 days. After tumors had grown to the approved size, all mice were sacrificed. Then the tumors were removed and weighed. As displayed in Figure 1E,F the volumes and weights of tumors were prominent decreased in the miR-92a-3p I group in comparison with the NC group.

### 2.2. Isolation and Identification of Glioma Stem-Like Cells (GSCs)

**Figure 2:**
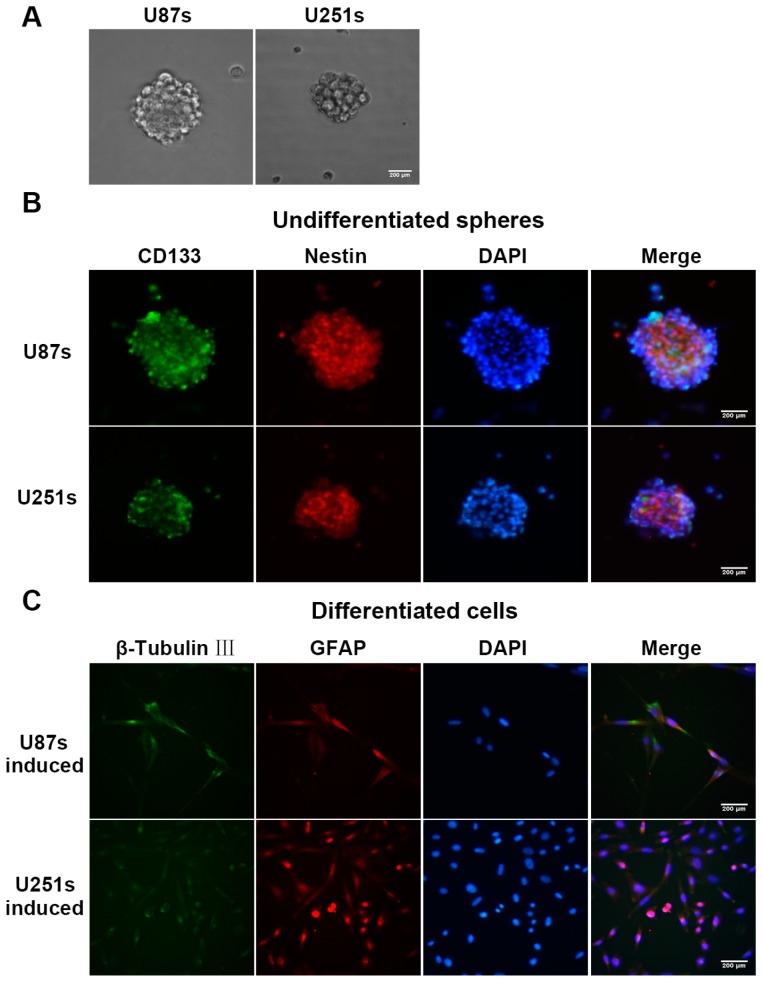
Isolation and identification of GSCs. (**A**) Representative images of primary GSCs; (**B**) Representative immunofluorescence staining images of CD133 (green) and nestin (red) in GSCs; (**C**) Representative immunofluorescence staining images of GFAP (red) and β-tubulin-III (green) in differentiated cells. Nuclei were counterstained with DAPI (blue) as a control. Images were photographed at 100× original magnification. All images are representative of three independent experiments images. Scale bar represents 200 µm.

The finding that knockdown of miR-92a-3p suppresses the malignancy of glioma cells raised the possibility that it may control the stem-like trait of GSCs. We enriched these cells from glioma cells by using serum-free culturing technology. After 7–10 days in culture, tumor spheres were visible under microscopy (Figure 2A). The images of immunofluorescence staining demonstrated that the cancer stem-like cell markers CD133 (green) and nestin (red) were expressed on the membranes of tumor spheres (Figure 2B). In the differentiation assay, tumor spheres were differentiated and display immunoreactivity for astrocyte’s maker glial fibrillary acidic protein (GFAP, red) and neuron marker β-tubulin-III (green) (Figure 2C). These data indicated that the isolated GSCs had the potential for multi-differentiation.

### 2.3. Over-Expression of miR-92a-3p Suppresses the Self-Renewal Ablity of Glioma Stem-Like Cells

**Figure 3:**
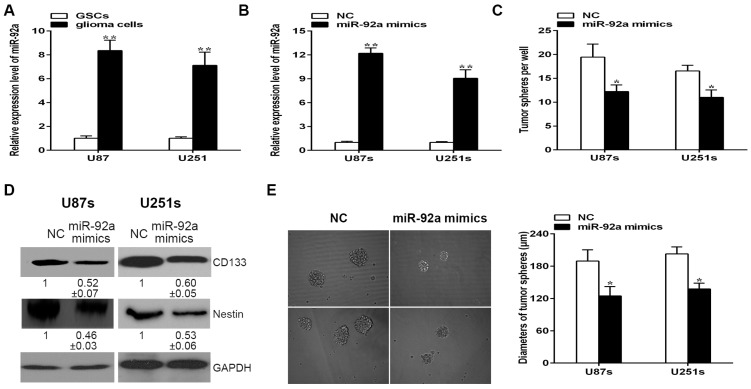
Function of miR-92a on the stemness of GSCs. (**A**) qRT-PCR analysis of miR-92a expression between glioma cells and GSCs; (**B**) qRT-PCR analysis of the expression of miR-92a in GSCs after treatment transfection with miR-92a mimics; (**C**) The self-renewal capability of GSCs was analyzed by tumor sphere formation assay; (**D**) The expression levels of cancer stem cell markers CD133 and Nestin were assessed by western blotting; (**E**) Diameters of GSCs were recorded and analyzed. Images were photographed at 100× original magnification. Each bar represents the mean of three independent experiments. * indicates *p* < 0.05, ** indicates *p* < 0.01. Scale bar represents 200 µm.

In an effort to examine the function of miR-92a-3p on GSCs, qRT-PCR analysis was used to investigate the miR-92a-3p expression pattern in glioma cells and GSCs. Interestingly, miR-92a-3p expression level was significantly over-expressed in glioma cells compared with GSCs (Figure 3A). We further estimate the function of miR-92a-3p on the self-renewal capability of GSCs. First of all, we confirmed that miR-92a-3p expression in glioma stem-like cell lines U87s and U251s were increased after miR-92a-3p mimics transfection (Figure 3B). Then the self-renewal ability of GSCs was evaluated by means of tumor sphere formation assay. The results indicated that overexpression of miR-92a-3p in GSCs impaired their ability to form tumor spheres (Figure 3C). In addition, elevating of miR-92a also resulted in a marked reduction in the expression of cancer stem-like cells marker CD133 and Nestin (Figure 3D), which is consistent with the inhibitory effect of miR-92a-3p on the tumor spheres formation capability of GSCs. Moreover, tumor spheres displayed a slightly decreased diameter post-treated with miR-92a-3p mimics compared with the NC group (Figure 3E). The above results revealed that miR-92a-3p could diminish the stemness of GSCs as a tumor suppressor.

### 2.4. miR-92a-3p Targets CDH1 in Glioma Cells While Bingding to the 3’UTR of Notch-1 in GSCs

**Figure 4:**
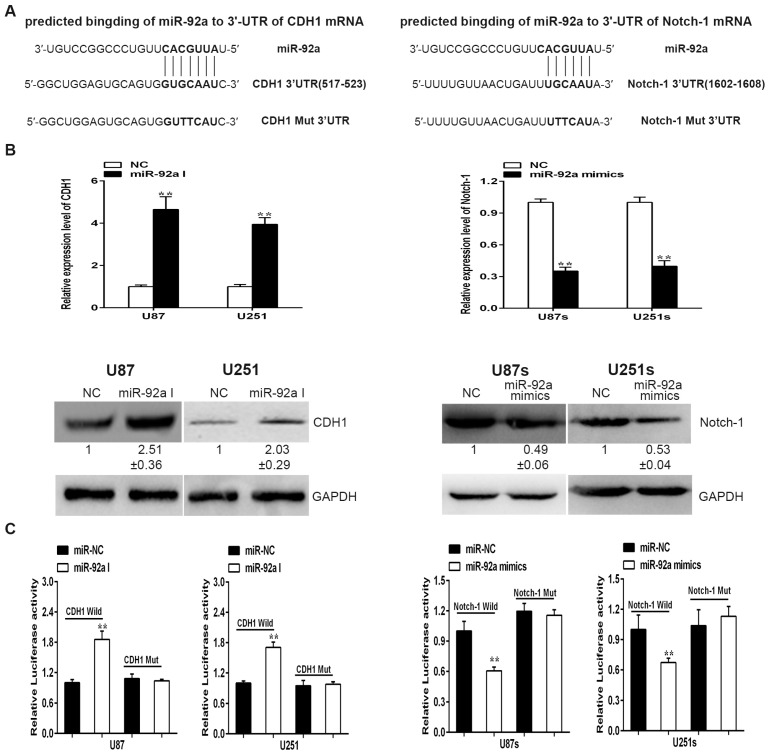
CDH1 and Notch-1 are direct targets of miR-92a-3p in glioma cells and GSCs, respectively. (**A**) Targeting sequences and mutated nucleotides of miR-92a-3p in 3′ UTR of CDH1 and Notch-1; (**B**) The mRNA and protein expression levels of CDH1 in glioma cells and Nocth-1 in GSCs were evaluated by qRT-PCR and Western blotting; (**C**) Relative luciferase activity of wild-type 3′ UTR and mutant type 3′ UTR of CDH1 and Notch-1. Each bar represents the mean of three independent experiments. ** indicates *p* < 0.01.

How does miR-92a-3p exert conversed effects in glioma cells and GSCs? Previous studies reported that miRNA could bind and silence hundreds of mRNA targets in various microenvironments [19,20], we wonder whether the different effect of miR-92a-3p in two cell types is a multi-target result. To confirm this possibility, we integrated bioinformatics analysis and mRNA and protein expression profiling to detect the downstream targets of miR-92a-3p. Numerous downstream targets were predicted by Targetscan and microRNA.org. By overlapping the lists of miR-92a-3p’s targets generated by two prediction tools, we narrowed the candidate targets of miR-92a-3p down to 10 genes (Table S1) mostly involved in cell proliferation, migration, invasion, or self-renewal ability of stem cells. Among these genes, CDH1 and Notch-1 (Figure 4A) are regarded as the regulators of Wnt/β-catenin and PI3K/Akt signaling pathways, respectively [21,22]. As the two pathways are always activated in glioma cells and GSCs [23,24,25,26], we hypothesize that CDH1 and Nocth-1 could be the direct targets of miR-92a. To test our hypothesis, qRT-PCR and Western blotting assay were applied to measure the relative expression levels of CDH1 and Notch-1 in two cell types. The data suggested that down-regulation of miR-92a-3p result in a robust increase of mRNA and protein levels of CDH1 in glioma cell, whereas up-regulation of miR-92a-3p induced a considerable decrease in the mRNA and protein levels of Notch-1in GSCs (Figure 4B). Interestingly, the CDH1 expression was positive correlated with the expression of miR-92a-3p in GSCs. Similarly, the expression pattern of Notch-1 was also consistent with miR-92a-3p in glioma cells (Figure S1), which indicated that miR-92a-3p may regulate different targets in glioma cells and GSCs. Furthermore, luciferase reporter plasmids of the wild-type or mutant 3′ UTR region of CDH1 and Notch-1 were conducted and co-transfected with miR-92a-3p I and miR-92a-3p mimics, respectively. The luciferase activity of wild-type 3′ UTR of CDH1 was much greater in the miR-92a-3p I group than that of the NC group. Meanwhile, the Luciferase intensity of wild type 3′ UTR of Notch-1 was lower when co-transfected with miR-92a-3p mimics in GSCs. However, the luciferase reporter expression with the mutant 3′ UTR of CDH1 and Notch-1 was not changed (Figure 4C). Together, these results implied that miR-92a-3p could directly acts on CDH1 or Notch-1 in a context-dependent manner.

### 2.5. miR-92a-3p Affected Glioma and GSCs Differently by Acting on CDH1/β-Catenin and Notch-1/Akt Signaling Pathways

**Figure 5:**
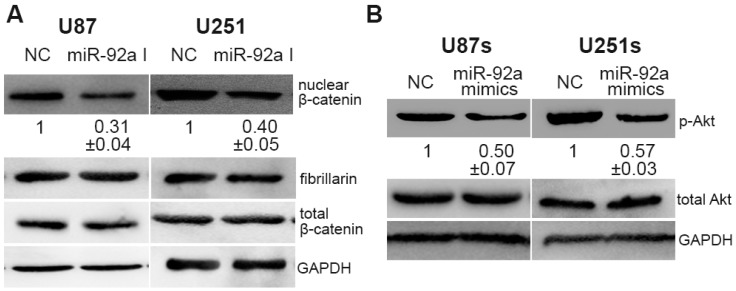
miR-92a-3p regulates CDH1/β-catenin signaling in glioma cells while affecting Notch-1/Akt signaling in GSCs. (**A**) Western blot analysis of total β-catenin and nuclear β-catenin expression levels in glioma cells post-treated with miR-92a-3p I; (**B**) Western blot analysis of total-Akt and p-Akt expression levels in GSCs after treated with miR-92-3p a mimics.

Functional validation of CDH1 and Notch-1 are the crucial effectors for miR-92a-3p prompted us to investigate additional downstream effectors of miR-92a-3p in two cell types. Previous studies denote that CDH1 is a negative regulator of the translocation of β-catenin from the cell membrane to the cytoplasm and nucleus, which has been implicated in cancer development and progression [21]. More important, Li et al. demonstrated that Akt is required for Notch-1-facilitated malignant behavior in breast cancer cells [22]. Therefore, we proposed that CDH1/β-catenin and Notch-1/Akt may be the downstream signaling pathway of miR-92a-3p in two cell types. Western blotting results showed that decreasing miR-92a-3p represses the protein level of nuclear β-catenin, whereas the total expression level of β-catenin remains unchanged in glioma cells (Figure 5A). As illustrated in Figure 5B, increasing miR-92a-3p reduced the phosphorylation level of Akt in GSCs. In contrast, no significant effect was found on total Akt. Collectively, these data demonstrated that miR-92a-3p targeted CDH1/β-catenin and Notch-1/Akt signaling pathways in glioma cells and GSCs, respectively.

### 2.6. CDH1and Notch-1 Is Required for miR-92a-3p to Exert Various Effects in Gliomas and GSCs, Respectively

**Figure 6:**
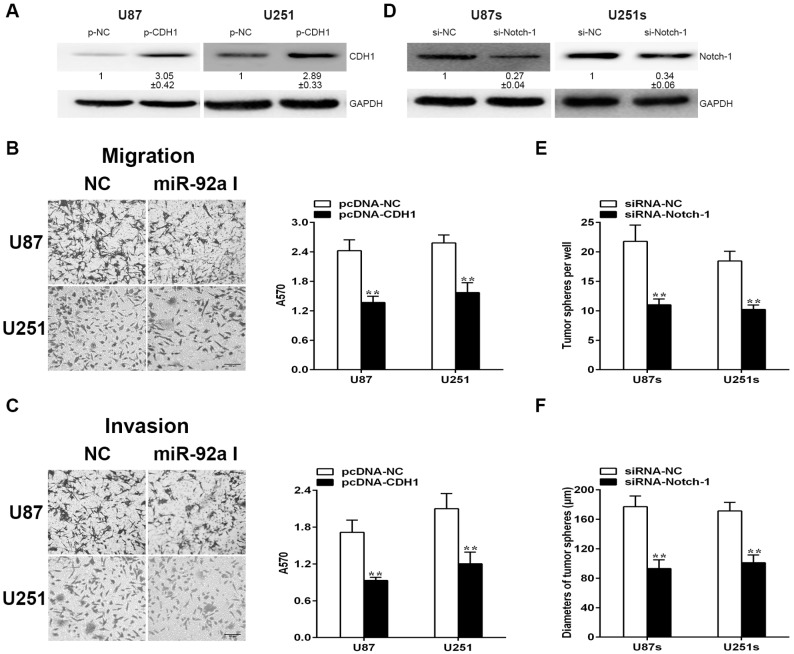
miR-92a-3p plays different roles in glioma cells and GSCs through targeting CDH1 and Notch-1, respectively. (**A**) Protein expressions of CDH1 in gliomas were measured by Western blotting; (**B**,**C**) Migration and invasion ability of glioma cells were detected through applying Transwell assay; (**D**) Protein expressions of Notch-1 in GSCs were qualified via Western blotting; (**E**,**F**) Stem-like traits of GSCs were evaluated by tumor sphere formation assay and diameters of GSCs. Each bar represents the mean of three independent experiments. ** indicates *p* < 0.01. Scale bar represents 200 µm.

Further we wonder whether the conversed function of miR-92a-3p is mediated by CDH1 and Notch-1. In glioma cells, CDH1 expression was elevated after being transfected with an over-expressed plasmid containing only the coding region of CDH1 (Figure 6A). As a consequence, the migration and invasion ability was markedly repressed, which is consistent with the effect of miR-92a-3p I on glioma cells (Figure 6B,C). However, little effect was found on cell proliferation (Figure S2). In addition, we used specific siRNA to suppress the expression of Notch-1 in GSCs (Figure 6D). As shown in Figure 6E,F, down-regulation of Notch-1 could mimic the suppression effect of miR-92a-3p mimics on the stem-like traits of GSCs. These results strongly suggested that CDH1 and Notch-1 play an essential effect in the regulation of miR-92a-3p on glioma cells and GSCs, respectively.

## 3. Discussion

A growing amount of evidence has shown that miRNAs play a critical role in the biological processes of cancer cells though targeting multiple downstream genes [10,11]. Recently, miR-92a-3p, a highly conserved microRNA, was reported to predominantly express in colorectal carcinoma [27], hepatocellular carcinoma [28], and esophageal squamous cell carcinoma [29]. In addition to its involvement in tumor development, miR-92a-3p has been found to be a regulator of human embryonic stem cell differentiation [30], suggesting that miR-92a-3p may be associated with the maintenance of cell stemness. In the present study, we observed that depression of miR-92a-3p suppressed the malignancy of glioma cells in vitro and in vivo, which are in consistent with the results that miR-92a-3p promoted the apoptosis of glioma cells [18]. However, the expression level of miR-92a-3p was reduced in GSCs when compared with it in glioma cells. Furthermore, increasing miR-92a-3p significantly inhibits the self-renewal capability of GSCs. Interestingly, these results demonstrated that miR-92a-3p could serve as either an onco-miR or a tumor suppressor or in different cell types. Of note, the contrary function of one specific miRNA in various cellular microenvironments has been published by other groups. For example, miR-125b accelerates the tumorgenesis of hematologic malignancies, but acts as a tumor suppressor in various solid tumors [31,32]. Moreover, Beatriz et al. [33] proposed that miR-221, miR-222, and miR-21 exerted opposite effects in glioma cells and GSCs. This apparent paradox might be contributed to the basis that a miRNA molecular has the ability to target different downstream mRNAs in various contexts, some of which may have inversed functions. Our data suggested that the suppression of a single miRNA for the purpose to improve glioma patients’ survival may not have the desired effects on averting glioma recurrence, which should be taken into consideration in future studies.

In an effort to elucidate the underlying molecular mechanism of miR-92a-3p in glioma cells and GSCs, we applied bioinformatics and predicted that CDH1 and Notch-1 are the potential target genes of miR-92a-3p. In addition, a markedly negative correlation between the expression of miR-92a-3p and CDH1 and Notch-1 was found in glioma cells and GSCs specifically. Further we identified that miR-92a-3p targeted CDH1 and Notch-1 in these cell types, respectively.

CDH1, a tumor suppressor in different kind of cancers, is a pivotal protein in maintaining cell to cell junctions to hold the epithelial cells tight, collectively, through anchoring β-catenin to the cell membrane [34,35]. Decreased expression of CDH1 and subsequent nuclear translocation of β-catenin compromised cellular adhesion and increased cellular invasive and metastatic potential in several types of cancers [36,37,38]. β-catenin is the central molecule effecter in the Wnt signaling pathway, which is highly conserved and regulates crucial steps in cancer pathologies [21,39]. Accumulation of nuclear β-catenin is a key event in the activation of the Wnt signaling pathway. By interaction with downstream transcription factors, such as TCF4 or Lef-1, the β-catenin/Lef/TCF complex was formed which impacts the transcription activity of multiple genes that are involved in cancer survival, metastasis, and apoptosis [21]. Recently, Ma et al. [40] demonstrated that, by directly targeting CDH1, miR-9 regulated the delivery of β-catenin to induce cellular metastasis in breast cancer. Additionally, miR-BART9 could also activate β-catenin through CDH1 and promote tumor metastasis in nasopharyngeal carcinoma [41]. In line with previous studies, our findings revealed that knockdown of miR-92a-3p led to a marked increase of CDH1 and a subsequent decrease of the expression level of nuclear β-catenin, while the total protein level of it remains unchanged. Furthermore, forced expression of CDH1 could mimic the inhibitory role of miR-92a-3p in glioma cell metastasis. These data suggested that the negative regulation of miR-92a-3p I on the migration and invasion ability of glioma cells was partly by blocking CDH-1/β-catenin signaling pathway.

Notch proteins (Notch-1–Notch-4) are an evolutionally-conserved family of trans-membrane receptors which regulate cell fate, stem cell self-renewal, and differentiation during development [42,43]. Notch-1 was linked to diverse cellular processes including cell proliferation, apoptosis, and cancer metastasis and angiogenesis [44]. Recently, Notch-1 was reported to take part in the regulation of malignant behaviors of breast cancer stem-like cells [45]. Moreover, Xu et al. [46] demonstrated that by interacting with the Akt signaling pathways, Notch-1 is involved in the growth and tumorgenesis of GSCs in vitro and in vivo. Likewise, in our study, Western blotting analysis demonstrated that the expression of Notch-1 and Akt phosophorylation were reduced significantly in GSCs post-transfection of miR-92a-3p mimics. However, no significant change was observed on the total level of Akt. Reduction of Notch-1 by specific siRNA could also inhibit the self-renewal ability of GSCs, suggesting that miR-92a-3p affects the maintenance of stemness of GSCs by down-regulating Notch-1 expression.

Besides, there are still a few limitations in the present study. The GSCs used in our research were derived from established cell lines. As the microenvironments of passaged cells might be different from primary tumor cells during long term passaged, the morphology and function of primary glioma cells were more similar to GBM than passaged cell lines. Therefore, these results need to confirm in vivo by further research.

## 4. Materials and Methods

### 4.1. Cell Culture

Human U87 and U251 glioma cells obtained from the Cell Bank of Chinese Academy of Sciences (Shanghai, China) were grown in Dulbecco’s Modified Eagle Medium (DMEM; Hyclone, Logan, UT, USA) supplemented with 10% Fetal Bovine Serum (FBS; Gibco, Carlsbad, CA, USA) at 37 °C in a humidified atmosphere of 5% CO_2_. The glioma stem-like cell lines U87s and U251s were isolated from U87 and U251 cells by using a serum-free clone formation method [14,47]. Glioma cells were dispersed and resuspended in the serum-free medium (SFM) composed of DMEM/F12 medium (Hyclone), B27 (20 mg/mL, Invitrogen, Carlsbad, CA, USA), 20 ng/mL basic fibroblast growth factor (bFGF; Sigma, St. Louis, MO, USA), 20 ng/mL EGF (PeproTech, Rocky Hill, NJ, USA). The primary tumor spheres were detected within 10 days of culture and subsequently dissociated and passaged in fresh medium every 3–4 days.

### 4.2. Differentiation Assay of Tumor Spheres

Tumor spheres were placed on poly-l-ornithine (BD Biosciences, San Diego, CA, USA) coated glass coverslips in cultural medium supplemented with 10% FBS. The coverslips were processed seven days after plating using immunocytochemistry. For immunofluorescence staining of differentiated tumor spheres, cells were double-stained with β-tubulin III (1:200, mouse monoclonal, Abcam, Cambridge, UK) and GFAP (1:200, rabbit polyclonal, Abcam) antibodies. For undifferentiated tumor spheres, cells were double-stained with CD133 (1:100, mouse monoclonal, Santa Cruz Biotechnology, Santa Cruz, CA, USA) and Nestin (1:100, rabbit polyclonal, Santa Cruz Biotechnology) antibodies. After that, the secondary antibodies (1:500, Fluorescein isothiocyanate (FITC)-conjugated goat anti-mouse or Texas red-conjugated goat anti-rabbit, Santa Cruz Biotechnology) were added. Then cells were counterstained with 4′,6-diamidino-2-phenylindole (DAPI, Invitrogen) to identify the nuclei. All images were photographed using a fluorescence microscope (Olympus, Lake Success, NY, USA).

### 4.3. Oligonucleotides, Plasmid and Transfection

The miR-92a-3p inhibitor (miR-92a-3p I), negative control (NC) inhibitor, miR-92a-3p mimics, negative control (NC) mimics, Notch-1 siRNA, and negative control siRNA (siRNA-NC) were chemically synthesized by GenePharma (Shanghai, China). The details were provided in Table 1. The CDH1 over-expression plasmid was a gift from Barry Gumbiner (Addgene plasmid # 45769) [48]. All transfections were conducted using Lipofectamine 2000 Transfection Reagent (Invitrogen) according to the manufacturer’s instructions.

### 4.4. RNA Isolation and Quantitative RT-PCR

Total RNA was extracted using TRIzol reagent (Invitrogen) in accordance with the standard protocol. With the help of a Nanodrop Spectrophotometer (ND-100, Thermo, Waltham, MA, USA), the RNA concentration was measured. cDNA from the total RNA was reverse transcripted using a HiScript 1st Strand cDNA Synthesis Kit (Vazyme, Nanjing, China) in accordance with the manufacturer’s protocol. cDNA from miRNAs was generated using a stem-loop RT-qPCR method. Quantitative real-time PCR was reacted to in triplicate in ABI StepOnePlusTM real-time PCR system (Applied Biosystems, Foster City, CA, USA). U6 and β-Actin were applied as endogenous controls for miRNA and mRNA expression profiles, respectively. Expressions were normalized to endogenous controls, then fold change in relative gene expression was calculated as 2^−ΔΔ*C*t^. The sequences of primers are listed in Table 2.

### 4.5. Cell Proliferation Assay

Following transfection with the miR-92a-3p I, MTT assay was applied to detect the proliferation of glioma cells. Briefly, cells were inoculated in a 96-well plates at the density of 2000 cells per well for each condition. Then the MTT (30 µL, 5 mg/mL, Sigma, St. Louis, MO, USA) was added to the medium and cultured for four hours. Finally, the supernatant was vacuumed and 150 µL Dimethyl Sulphoxide (DMSO, Sigma) was used to dissolve the formazan crystals into each well. The absorbance at 490 nm was detected with a SpectraMax M5 microplate reader (Molecular Devices, Silicon Valley, CA, USA).

### 4.6. Cell Migration and Invasion Assay

Transwell filters (8 µm pore size, Millipore, Billerica, MA, USA) coated with or without Matrigel (BD Biosciences) were performed to evaluate the metastasis of glioma cells. The transfected cells (2 × 10^5^) were resuspended in serum-free medium and plated in the upper filter. A DMEM medium supplemented with 10% FBS was added in the bottom filter. After incubated for 48 h, by using a cotton swab, cells on the upper membrane were mechanically scraped. Cells on the lower side of the filter were fixed with 100% methanol (Sigma) for 10 min, dried and stained with 1% crystal violet (Sigma) for 20 min. Three independent fields of cells adhered to the lower side of transewll filters were photographed for each well. Then stained cells were dissolved in 33% acetic acid (Sigma). And the absorbance at 570 nm was recorded to evaluate the migration ability.

### 4.7. Tumor Sphere Formation Assay

GSCs were dissociated to single cells and transfected with miR-92a-3p mimics or negative control mimics as described above. Then cells were seeded at a density of 200 cells per well in 24-well plates and the medium was refreshed every two days. The numbers of tumor spheres per well was recorded and the tumor sphere formation rate was calculated on day 7.

### 4.8. Target Prediction

Two bioinformatics algorithms: TargetScan Human Release 6.2 [49] and Microrna.org (August 2010 Release) [50] were used to predict the potential target genes of miR-92a-3p. TargetScan ranked the predicted targets by cumulative weighted context++ score in descending order while Microrna.org arranged genes by mirSVR score in ascending order.

### 4.9. Reporter Vectors Construction and Luciferase Reporter Assay

The wild-type or mutant CDH1/Notch-1 3′ UTR luciferase reporter containing the predicted binding site for miR-92a-3p was chemical synthesized and cloned to the pGL3-Control Vector (Promega, Madison, WI, USA). Glioma cells or GSCs were cultured in 96-well plates and co-transfected with wild or mutant luciferase reporter vectors and miR-92a-3p inhibitor or negative control inhibitor using Lipofectamine 2000 (Invitrogen). Following 48 h incubation, the luciferase activity was quantified using a dual-luciferase reporter system (Promega). Renilla luciferase activity was used as an endogenous controls.

### 4.10. Western Blot Analysis

Western blot analysis was performed as standard procedures previously described [41]. The following primary antibodies directed against the antigens were used: CDH1 (1:1000, mouse monoclonal, Cell Signaling, Danvers, MA, USA), fibrillarin (1:2000, rabbit monoclonal, Cell Signaling), β-catenin (1:2000, rabbit monoclonal, Cell Signaling), Notch-1 (1:1000, rabbit polyclonal, Santa Cruz Biotechnology), p-Akt-T308 (1:1000, rabbit monoclonal, Cell Signaling), total-Akt (1:2000, mouse monoclonal, Cell Signaling), glyceraldehyde-3-phosphate dehydrogenase (GAPDH, 1:5000, Vazyme). GAPDH was used as the loading control in the Western blots.

### 4.11. Animal Studies

U87 glioma cells were implanted into the right and left flanks (1.0 × 10^7^ cells per flank) of four-week-old female nude mice (Model Animal Research Center of Nanjing University, Nanjing, China). When the tumor volume reached about 100 mm^3^, all nude mice were treated with 200 pmol miR-92a-3p I or scramble oligo combined with 10 µL Lipofectamine 2000 via local injection of the established xenograft tumor once every two days for 28 days. The tumor volume was calculated by using a caliper every two days with the formula: volume = (length × width^2^)/2. Tumor formation in mice was recorded by measuring tumor weight later. This study was approved by the Ethics Committee of the Medical School of Southeast University (201509080012, 08 Sep 2015) and all animal experiments were treated in accordance with the guidelines for the care and use of laboratory animals of National Institutes of Health [51].

### 4.12. Statistical Analysis

All tests were performed using GraphPad Prism 6 software (GraphPad Software, Inc., La Jolla, CA, USA). All data are shown as the mean ± S.D. Data were analyzed using the two-tailed Student *t*-test (for two samples) or One-way Analysis of Variance (ANOVA, for multiple samples). *p* < 0.05 was considered statistically significant.

## 5. Conclusions

In summary, our current results reported the multiple functions of miR-92a-3p on the malignancy of glioma cells and self-renewal of GSCs for the first time. miR-92a-3p controlled these phenotypes by targeting CDH1 and Notch-1 and subsequently affected the β-catenin and Akt signaling pathways in a context-dependent manner. These data revealed that miR-92a-3p may play critical roles in the development of glioma.

## Figures and Tables

**Table 1:** Sequence of oligonucleotides used in this study.

Oligonucleotides	Sequence 5′ to 3′
miR-92a-3p inhibitor	ACAGGCCGGGACAAGUGCAAUA
negative control inhibitor	UCUACUCUUUCUAGGAGGUUGUGA
miR-92a-3p mimics	UAUUGCACUUGUCCCGGCCUGU
negative control mimics	UCACAACCUCCUAGAAAGAGUAGA
Notch-1 siRNA	UGGCGGGAAGUGUGAAGCG
negative control siRNA	UUCUCCGAACGUGUCACGU

**Table 2:** Primers used for qRT-PCR.

Gene	Primer	Sequence 5′ to 3′
*miR-92-3p*	RT	GTCGTATCCAGTGCAGGGTCCGAGGTATTCGCACTGGATACGACACAGGC
	Forward	GGGGCAGTTATTGCACTTGTC
	Reverse	CCAGTGCAGGGTCCGAGGTA
*U6*	RT	AAAAATATGGAACGCTCACGAATTTG
	Forward	GTGCTCGCTTCGGCAGCACATATAC
	Reverse	AAAAATATGGAACGCTCACGAATTTG
*CDH1*	Forward	AAAGGCCCATTTCCTAAAAACCT
	Reverse	TGCGTTCTCTATCCAGAGGCT
*Notch-1*	Forward	GAGGCGTGGCAGACTATGC
	Reverse	CTTGTACTCCGTCAGCGTGA
*β-Actin*	Forward	AAAGACCTGTACGCCAACAC
	Reverse	GTCATACTCCTGCTTGCTGAT

## Supplementary Materials

Supplementary materials can be found at www.mdpi.com/1422-0067/17/11/1799/s1.
