# Supplementary material for: miR-92a-3p Exerts Various Effects in Glioma and Glioma Stem-Like Cells Specifically Targeting CDH1/β-Catenin and Notch-1/Akt Signaling Pathways
Source: Int J Mol Sci. 2016 Oct 27;17(11):1799. doi: 10.3390/ijms17111799 (PMC5133800; doi:10.3390/ijms17111799)
Supplement: Supplementary file 1 [file ijms-17-01799-s001.pdf]

Supplementary Material: miR-92a-3p Exerts Various Effects in Glioma and Glioma Stem-Like Cells Specifically Targeting CDH1/β-Catenin and Notch-1/Akt Signaling Pathways

Hang Song, Yao Zhang, Na Liu, Sheng Zhao, Yan Kong and Liudi Yuan

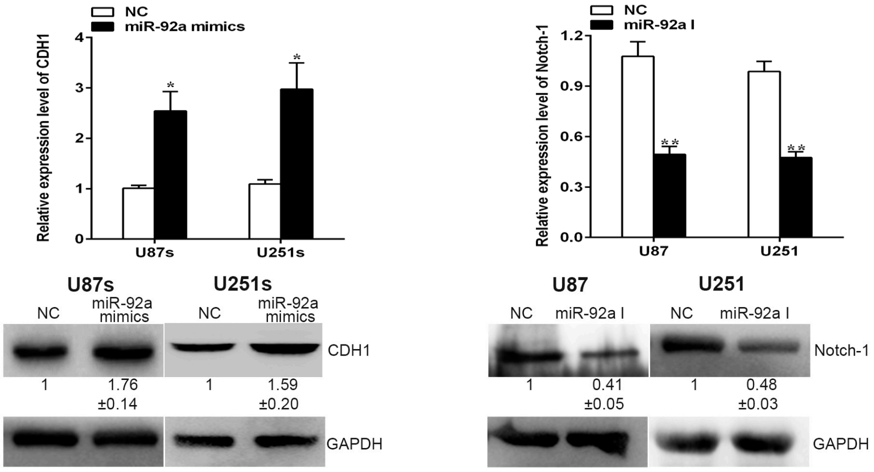

Figure S1. The mRNA and protein expression levels of CDH1 and Notch-1 were positively correlated with miR-92a in GSCs and glioma cells, respectively. * indicates $p < 0.05$, ** indicates $p < 0.01$.

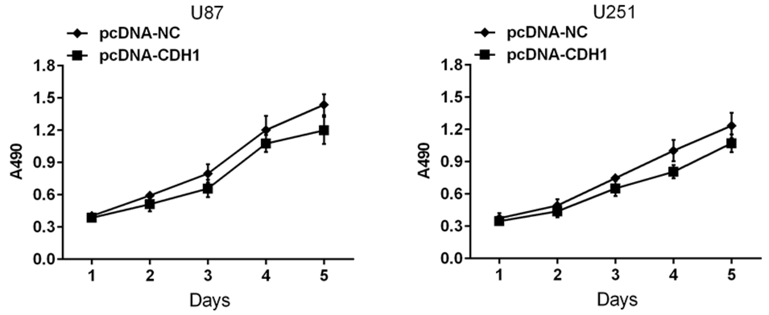

Figure S2. The effect of CDH1 on the proliferation of glioma cells in vitro.

Table S1. Top 10 miR-92a-3p's targets predicted by Targetscan and Microrna.org. These genes are mostly involved in cell proliferation, migration, invasion, or self-renewal ability of stem cells.

MicroRNA	Gene ID	Gene
miR-92a-3p	1,0114	*HIPK3*
miR-92a-3p	1,0140	*TOB1*
miR-92a-3p	1028	*CDKN1C*
miR-92a-3p	999	*CDH1*
miR-92a-3p	1,0365	*KLF2*
miR-92a-3p	4581	*NOTCH-1*
miR-92a-3p	1,0579	*TACC2*
miR-92a-3p	109	*ADCY3*
miR-92a-3p	1008	*CDH10*
miR-92a-3p	1,0018	*BCL2L11*
